# Compression of the Fourth Ventricle Using a Craniosacral Osteopathic Technique: A Systematic Review of the Clinical Evidence

**DOI:** 10.1155/2017/2974962

**Published:** 2017-10-18

**Authors:** Anna Żurowska, Roksana Malak, Anna Kołcz-Trzęsicka, Włodzimierz Samborski, Małgorzata Paprocka-Borowicz

**Affiliations:** ^1^Department of Physiotherapy, Faculty of Health Sciences, Wrocław Medical University, 2 Grunwaldzka Street, 50-355 Wrocław, Poland; ^2^Department of Rheumatology and Rehabilitation, Faculty of Health Sciences, Poznan University of Medical Sciences, 135/147 28 Czerwca 1956 Street, 61-545 Poznań, Poland

## Abstract

Compression of the fourth ventricle (CV4) is a well-known osteopathic procedure, utilized by osteopaths, osteopathic physicians, craniosacral therapists, physical therapists, and manual therapists as part of their healthcare practice based on some evidence suggesting impact on nervous system functions. The main objective of the study was to identify randomized controlled trials (RCTs) assessing the clinical benefits of CV4 and to show the evidence supporting clinical prescriptions, guides, and advice in treating. A computerized search of the PubMed, CINAHL Complete, Scopus, Web of Science, and ScienceDirect databases was performed. Two filters were used (article type: RCTs; species: humans). The methodological quality of the trials was assessed using the Downs and Black quality checklist for healthcare intervention studies. Only six studies met the inclusion criteria, of which four were RCTs and two were observational studies. The Downs and Black score ranged from 17 to 24 points out of a maximum of 27 points. The present review revealed the paucity of CV4 research in patients with different clinical problems, as five out of six included studies investigated healthy adults. According to the results of the included studies, CV4 may be beneficial for patients with different functional problems.

## 1. Introduction

Compression of the fourth ventricle (CV4) is a well-known osteopathic procedure, which was first described by Sutherland, DO, in 1939 [1]. Nowadays, it is practiced by osteopaths, craniosacral therapists, physical therapists, and manual therapists [2–5]. CV4 is described as a cranial manipulation technique aimed at influencing the function of the nervous system, including the autonomic nervous system [6, 7]. Increased electroencephalography alpha absolute power, reduced sleep latency, and even decreased anxiety were shown in many studies as the effect of the technique [2]. Also, an effective and progressive cerebral hemodynamic response was shown [8]. The procedure of CV4 appears to be simple; the patient lays down, and the therapist holds a squamous part of the occipital bone with its lateral angles and manipulates the cranium into an extension. The therapist holds the extension of the cranium and waits for a motionless state. After the appearance of the cranial pulse, the therapist may end the procedure [6].

There are a number of researches which show the effectiveness of this technique. Referring to the benefits of CV4, cranial manipulation may modify the heart rate, blood flow velocity, blood pressure, and cerebral tissue oxygenation and reduce symptoms of otitis media and colic [4, 7]. It should be mentioned that CV4 may also influence the condition of children with cerebral palsy [9]. Patients with other neurologic disorders, such as adults with multiple sclerosis, have better function of the lower urinary tract after CV4 procedure, which was a component of the craniosacral therapy (CST) protocol [10]. One study also reported efficacy in the treatment of tension-type headache [11].

The main objective of the study was to identify randomized controlled trials assessing the clinical benefits of CV4 and to show evidence supporting clinical prescriptions, guides, and advice when treating.

## 2. Materials and Methods

Computerized searching of the following literature databases was performed from the initiation of the database until August 2017: PubMed, CINAHL Complete, Scopus, Web of Science, and ScienceDirect. The following combinations of keywords were used to search for the intervention of interest: “fourth cerebral ventricle”, “CV4”, “CV4 compression”, “compression of the fourth ventricle”, “CV4 compression manoeuvre”, “cranial osteopathic technique”, “craniosacral therapy”. The displayed items were screened for possible inclusion in this review. Two filters were used (article type: randomized controlled trials (RCTs), species: humans).

### 2.1. Inclusion and Exclusion Criteria

The following inclusion criteria were defined in this review: (1) study design, published RCTs; (2) population: studies conducted on human patients; (3) disease: no restrictions on the pathogenesis and etiology of the disease; (4) intervention: studies must report the CV4 technique as the only treatment provided, performed by an osteopath or craniosacral therapist and defined as CV4 by the authors themselves; (5) indication: studies must investigate the physiological effects of CV4 technique; (6) language restrictions: only originally English language papers were considered.

Exclusion criteria were as follows: (1) study design: non-RCTs; (2) population: animal models; (3) intervention: the CV4 technique combined with additional osteopathic or manipulative medicine treatments; (4) indication: lack of identification of the physiological effects of CV4 technique; (5) language restrictions: language other than English.

### 2.2. Citation Screening and Data Extraction

The papers were first screened based on the title and abstract supplied with each citation. Each paper was screened by two independent reviewers. All discrepancies between reviewers were discussed and resolved. First, papers that did not match the inclusion criteria were excluded. Full-text copies of all papers that could potentially meet the inclusion criteria were obtained at this stage. For each included study, the following data were extracted: (a) general study information (study size, study design, and practitioner profile), (b) participants data (treatment duration and description), and (c) outcomes (physiological parameters).

### 2.3. Quality Assessment

The Downs and Black quality checklist for healthcare intervention studies was used to evaluate articles included in the present review [16, 17]. Each article was assessed by three independent reviewers using this scoring system. The Downs and Black score was designed to evaluate the quality of original research articles. The checklist encompasses 27 “yes or no” questions that are organized into five sections: (1) study quality (10 items), assessing the overall quality of the study; (2) external validity (3 items), to determine the ability to generalize the findings of the study; (3) study bias (7 items), to assess bias in the intervention and outcome measure(s); (4) confounding and selection bias (6 items), to determine bias from sampling or group assignment; and (5) power of the study (1 item), to determine whether the findings are due to chance (National Collaborating Centre). Any discrepancies between reviewers were discussed and resolved.

## 3. Results

**Figure 1:**
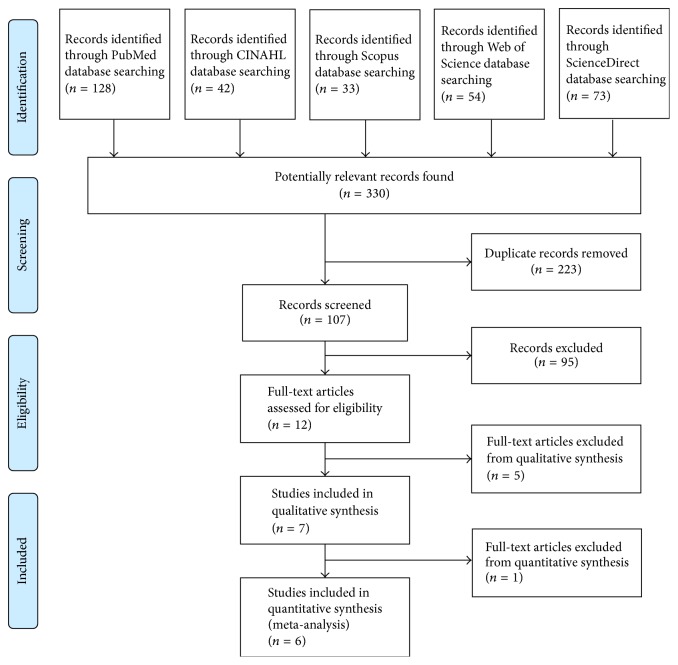
Flow chart of the review process according to the PRISMA (Preferred Reporting Items for Systematic Reviews and Meta-Analyses) guidelines.

Three hundred and thirty potentially relevant articles were found through the literature search. 128 of those were identified on PubMed, 42 on CINAHL Complete, 33 on Scopus, 54 on Web of Science, and 73 on ScienceDirect. Following the first abstract review, 12 potentially relevant articles were identified. At this stage, full-text versions were obtained for further evaluation. Following detailed evaluation, 7 articles that met the inclusion criteria were included in qualitative synthesis and 6 of them were qualified into meta-analysis (Figure 1).

### 3.1. Included Clinical Studies

Seven RCTs that met the inclusion criteria of this review examined the physiological effect of one craniosacral technique: compression of the CV4 (Table 1).

### 3.2. Trial Design Characteristics

Table 2 presents basic information, study design details, outcomes, and practitioners' competences.

### 3.3. Clinical Benefits

The most commonly used outcome measurements were various physiological parameters and autonomic nervous system (ANS) function [6, 12, 14], brain cortex activity (especially in alpha band), and pain (visual analog scale). One study investigated the influence of CV4 on sleep latency and revealed more rapid sleep onset and MSNA (muscle sympathetic nerve activity) decrease in comparison to prestillpoint after CV4 in the experimental group [12].

It was investigated that the influence of CV4 on pain level in patients with tension-type headaches showed a significant improvement [11]. In the previous study evaluating pre- and posttreatment changes in healthy adults, the significant influence of CV4 on the brain cortex activity in electroencephalography (EEG) recording was retrieved, and the alpha absolute power increase was shown [2]. Another EEG study in patients with low back pain (LBP) determined that the CV4 significantly modulates peak alpha frequency and promotes physical relaxation [13]. Out of the three studies [6, 12, 14] that investigated the influence of CV4 on ANS function, only one reported a significant decrease in systolic blood pressure [6] and one a decrease of muscle sympathetic nerve activity [12]. One study which examined the influence of CV4 on blood flow velocity reported that CV4 procedure especially affects the low-frequency oscillations in blood flow velocity. After application, the amplitude of the Traube–Hering–Mayer oscillation (THMO), 0.10 Hz, frequency wave increased [15]. A summary of outcomes is presented in Table 3.

### 3.4. Quality of Studies

The checklist score for each included study is reported in Table 4. Methodological Downs and Black quality scores ranged from 17 to 24 points out of a theoretical maximum of 27 points. Two studies gained a strong-quality rating (21 and 24 points) [11, 15]. Five remaining RCTs scored between 17 [2] and 19 [6, 12] points. The reporting and external validity was similar in all included studies. Internal validity (bias) was better in general than internal validity (confounding) (Table 4).

## 4. Discussion

Osteopaths use the osteopathic manipulative treatment (OMT) to treat somatic dysfunction, such as tissue modifications, impaired range of motion, and asymmetry. OMT has been claimed to produce anti-inflammatory and hyperparasympathetic effects [18]. OMT practitioners take advantage of several manual techniques and approaches, ranging from articulatory, fascial, or visceral manipulation to cranial osteopathy, which are considered the most popular [19, 20].

CST was developed in the 1970s and it is described as a gentle approach that releases tension deep in the body to relieve pain and dysfunction and improve whole-body health and performance. These techniques are able to release restrictions in the soft tissues that surround the central nervous system by using gentle touch [21, 22].

The systematic review showed that there are only a few studies suggesting the effectiveness of the CV4 therapy. The selection and criteria used helped identify ten research publications that presented the overall quality of the study. Some reported outcomes such as pain relief and autonomic nervous system regulation were statistically significant. However, other reported results such as blood pressure and heart rate regulation were described as the truly presented effects during and after the treatment. However, there were no statistically significant results [6, 12]. Patients with tension-type headache (TTH) may feel the relief after CV4. The examination of Hanten et al. [11] showed the effectiveness of treating TTH by CV4 in comparison to resting position techniques during pain. Low back pain was another condition in which CV4 seemed to play an important role as the analgesic technique [13].

Although pediatric patients were described in the introduction of the study as the population that may benefit from reducing abdominal pain and complex regional pain syndrome [23, 24] or even in conditions such as irritable bowel syndrome [25, 26], it is the adult population that was presented in the results as the population that had the most noticeable effects following CV4.

Regulation of the ANS parameters was described as a result of the CV4 procedure. Catecholamines and systolic blood pressure (SBP) and diastolic blood pressure (DBP) are important regulators of the ANS. However, no significant changes were shown referring to catecholamines. The increase in dopamine blood levels after the intervention was shown in some studies. Nonetheless, the results were not statistically significant [6]. The significant decrease in the intervention group was presented after treatment according to SBP and during the treatment referring to DBP, but with a nonsignificant tendency in the postintervention period [6].

Some researches emphasized the increase in parasympathetic activity after the CV4 technique; it is the sympathetic activity that might increase during the procedure [12]. Referring to parameters of the autonomic nervous system such as skin temperature, galvanic skin resistance, or heart rate variability, there might be minimal physiologic effects seen in any of the autonomic variables recorded. Despite the initial sympathetic skin response reported during the first minute of each phase, it was mentioned that galvanic skin resistance might differ depending on psychological and personality factors [12]. The balance of both systems should always be considered as theoretical principles emphasized by Chaitow [27, 28]. The expected effect of CV4 is based on a decrease in sympathetic activity and an increase in parasympathetic modulation during application of the technique. Respiration rate was not altered during the touch or the period of CV4 and also in the control group that received just touch instead of treatment.

The benefit of CV4 mentioned in the research was shown by EEG as changes in the alpha band activity in the occipital areas and as an indicator of physical relaxation [2]. The author of the study mentioned in the discussion that there are other researchers, for instance, Shi et al. [8], who demonstrated the relaxation effect of the CV4. However, they were not considered in the review study. Some results presented by Miana et al. [2] were not statistically significant, for example, differences between pre- and postabsolute power levels for the control and sham CV4 conditions. Other results of the analysis correlated with relaxation were sleep latency and muscle sympathetic nerve activity. Sleep latency decreased thanks to CV4 technique. The MSNA as a potential mechanism for altered sleep latency is considered as being decreased in comparison to the prestillpoint phase of CV4 [12].

Changes such as in the dynamics of cerebrospinal fluid lead to better perfusion of the cortical region [2].

It is clear that the CV4 technique may alter the physiologic parameters of blood flow velocity, heart rate, blood pressure, and cerebral tissue oxygenation. Not all parameters were statistically significant [15]. For instance, HR and BP were not significantly different at any time points during all three trials in the studies by Cutler et al. [12] and Cardoso-de-Mello-e-Mello-Ribeiro et al. [6]. However, the CV4 procedure specifically affected the low-frequency oscillations in blood flow velocity. After application, the amplitude of the THMO, the dominant 0.10 Hz frequency wave, increased, leading some authors to suggest that the cranial technique might be applied to alter blood flow during some interventional directives [15].

A limitation of the reviewed studies is that their subject populations consisted of healthy, asymptomatic individuals. It is possible that, had these studies been conducted on subjects with a common set of conditions, the significance of the results may have been augmented. The other limitation of this work is that the research groups were quite small and some of the studies did not consider a control group. Despite these limitations, this systematic review showed that CV4 technique may be beneficial, especially for adults with tension-type headaches or with low back pain. The cranial manipulation may be helpful if there is an imbalance in the autonomic nervous system which is common in many diseases.

The mechanisms of many manual techniques are not well known or studied. It is possible that CV4 influences the receptors in the occipital area (suboccipital muscles) [4]. Therefore, there is a need for more studies with larger groups, including subjects with pathological conditions and a control group, in order to assess the more widespread effects of CV4.

## 5. Conclusions

This systematic review provides the most recent update on the evidence of clinical benefits of CV4 in healthy adults, based on the studies available in two databases. The methodological quality of the studies did not change over the last ten years. The current moderate quality of the studies and insufficiency of available data, especially regarding the population of patients with different clinical problems, suggest that more research into this area is needed.

## Figures and Tables

**Table 1:** Characteristics of the included studies.

Study	Design	Objective of study
Cardoso-de-Mello-e-Mello-Ribeiro AP et al., 2015 [6]	RCT	To evaluate the effects of the fourth-ventricle compression on the autonomic nervous system.
Cutler et al., 2005 [12]	RCT	To determine whether cranial manipulation alters sleep latency and investigate its effects on MSNA as a potential mechanism for altered sleep latency.
Hanten et al., 1999 [11]	RCT	To investigate the effectiveness of CV4 and resting techniques, following single treatment, in patients with TTH.
Miana et al., 2013 [2]	RCT	To measure absolute alpha power in the occipital area in healthy adults.
Martins et al., 2015 [13]	RCT	To determine immediate changes in electroencephalography activity in individuals with nonspecific chronic LBP.
Milnes and Moran, 2007 [14]	Before-and-after study	To investigate the physiological effects of a single CV4 in comparison to simple touch.
Nelson et al., 2006 [15]	Before-and-after study	To examine the CV4 and its effect upon blood flow velocity.

RCT: randomized controlled trial; CV4: compression of the fourth ventricle; TTH: tension-type headache; MSNA: muscle sympathetic nerve activity; LBP: low back pain.

**Table 2:** Basic information including study design details, clinical outcomes, and practitioners' competences.

Study	Patient population	Follow-up	Assigned groups	*N*	Number of sessions/length of session	Practitioner profile	Type of intervention
Cardoso-de-Mello-e-Mello-Ribeiro et al., 2015 [6]	Healthy adults (physiotherapy students)	None	Intervention group Control group	20 20	10 min 10 min	Experienced physiotherapist	Compression of the CV4

Cutler et al., 2005 [12]	Healthy adults	None	1 group underwent randomly 3 procedures	20	Unknown	Unknown	CV4 CV4 sham No treatment

Hanten et al., 1999 [11]	Patients with episodic or chronic TTH	None	Control group Intervention 1 Intervention 2	60	10 min 10 min 10 min	3 investigators	No treatment Most comfortable position of head protraction/retraction and flexion/extension CV4

Miana et al., 2013 [2]	Healthy adults	None	1 group underwent randomly 3 procedures	10	10 min	Unknown	CV4 CV4 sham No treatment

Martins et al., 2015 [13]	Patients with chronic LBP	None	1 group underwent randomly 3 procedures	81	Unknown	Osteopath Physiotherapist 5th-year osteopathy student	CV4 CV4 sham No treatment

Milnes and Moran, 2007 [14]	Healthy adults	None	Intervention group	10	1–10 min 2–5 min 3, individual 4-5 min 5–10 min	Experienced osteopath	Phase 1: baseline Phase 2: touch Phase 3: CV4 Phase 4: touch Phase 5: baseline

Nelson et al., 2006 [15]	Healthy adults	None	Intervention group	26	5–7 min 1,5–10 min 5–7 min	Osteopath	Phase 1: baseline Phase 2: CV4 Phase 3: response

*N*: number of participants; CV4: compression of the fourth ventricle; TTH: tension-type headache; LBP: low back pain.

**Table 3:** Main study outcomes and effects in comparison to control patients or baseline results.

Study	Outcomes and methods used	Condition	Effect/result in comparison to control group and/or baseline
Cardoso-de-Mello-e-Mello-Ribeiro et al., 2015 [6]	BP, HR, plasmatic catecholamine levels	Healthy adults	Dopamine: no significant change Norepinephrine: no significant change Epinephrine: no significant change SBP: significant decrease in intervention group after treatment DBP: significantly lower in intervention group with nonsignificant tendency in postintervention comparison HR: nonsignificant lower values after intervention in comparison to baseline

Cutler et al., 2005 [12]	Multiple sleep latency test protocol, HR, BP, EEG, EOG, EMG, postganglionic MSNA micrography	Healthy adults	More rapid sleep onset in the CV4 treatment group MSNA: decrease in comparison to prestillpoint phase of CV4 HR and BP were not significantly different at any time points during all three trials

Hanten et al., 1999 [11]	Visual analog scale	Subjects with TTH	Significant improvement in the group treated with CV4 in comparison to the group with no treatment

Miana et al., 2013 [2]	EEG	Healthy adults	No significant differences between pre- and postabsolute power levels for the control and sham CV4 conditions Significant increase in the alpha absolute power in CV4 group when compared to moments before and after treatment

Martins et al., 2015 [13]	EEG	Subjects with LBP	Modulation of the brain cortex electrical activity measured by EEG mean change in the peak alpha frequency

Milnes and Moran, 2007 [14]	Galvanic skin resistance, skin temperature, HRV, respiration rate	Healthy adults	Application of the CV4 technique had a minimal physiologic effect (not significant) in the autonomic variables recorded

Nelson et al., 2006 [15]	Laser transcutaneous blood flow meter	Healthy adults	The CV4 procedure specifically affected the low-frequency oscillations in blood flow velocity; after application, the amplitude of the THMO, 0.10 Hz, frequency wave increased

BP: blood pressure; HR: heart rate; EEG: electroencephalography; EMG: electromyography; EOG: electrooculography; CV4: compression of the fourth ventricle; MSNA: muscle sympathetic nerve activity; TTH: tension-type headache; LBP: low back pain; THMO: Traube–Hering–Mayer oscillation; DBP: diastolic blood pressure; SBP: systolic blood pressure.

**Table 4:** Summary of critical appraisal scores of the included studies (Downs and Black checklist).

Study		Cardoso-de-Mello-e-Mello-Ribeiro et al., 2015 [6]	Cutler et al., 2005 [12]	Hanten et al., 1999 [11]	Miana et al., 2013 [2]	Milnes and Moran, 2007 [14]	Nelson et al., 2006 [15]
1	Reporting	Y	Y	Y	Y	Y	Y
2		Y	Y	Y	Y	Y	Y
3		Y	Y	Y	Y	Y	Y
4		Y	Y	Y	Y	Y	Y
5		N	N	Y	N	N	N
6		Y	Y	Y	Y	Y	Y
7		Y	Y	Y	Y	Y	Y
8		N	Y	N	N	U	Y
9		N	Y	Y	N	Y	Y
10		Y	Y	Y	Y	Y	Y

11	External validity	Y	U	U	Y	Y	Y
12		U	U	N	U	N	U
13		U	Y	U	U	N	Y
14		Y	Y	Y	Y	Y	Y
15		Y	Y	Y	Y	Y	Y
16		Y	Y	Y	Y	Y	Y
17		Y	Y	Y	Y	Y	Y
18		Y	Y	Y	Y	Y	Y
19		Y	Y	Y	Y	U	Y
20		Y	Y	Y	Y	Y	Y
21		Y	U	Y	U	Y	Y

22	Confounding	U	N	U	U	U	Y
23		U	N	U	U	U	Y
24		U	N	Y	U	U	Y
25		Y	U	Y	U	U	N
26		Y	Y	Y	Y	Y	Y

27		Y	Y	Y	Y	Y	Y

*Total*		*19*	*19*	*21*	*17*	*18*	*24*

Y: yes; N: no; U: unable to be determined.

## References

[1] Sutherland W. G. (1939). *The Cranial Bowl*.

[2] Miana L., Hugo do Vale Bastos V., Machado S. (2013). Changes in alpha band activity associated with application of the compression of fourth ventricular (CV-4) osteopathic procedure: a qEEG pilot study. *Journal of Bodywork and Movement Therapies*.

[3] Jäkel A., von Hauenschild P. (2012). A systematic review to evaluate the clinical benefits of craniosacral therapy. *Complementary Therapies in Medicine*.

[4] Ferguson A. (2003). A review of the physiology of cranial osteopathy. *Journal of Osteopathic Medicine*.

[5] Matarán-Peñarrocha G. A., Castro-Sánchez A. M., García G. C., Moreno-Lorenzo C., Carreño T. P., Zafra M. D. O. (2011). Influence of craniosacral therapy on anxiety, depression and quality of life in patients with fibromyalgia. *Evidence-Based Complementary and Alternative Medicine*.

[6] Cardoso-de-Mello-e-Mello-Ribeiro A. P., Rodríguez-Blanco C., Riquelme-Agulló I., Heredia-Rizo A. M., Ricard F., Oliva-Pascual-Vaca Á. (2015). Effects of the fourth ventricle compression in the regulation of the autonomic nervous system: a randomized control trial. *Evidence-Based Complementary and Alternative Medicine*.

[7] Malliani A. (2006). Cardiovascular variability is/is not an index of autonomic control of circulation. *Journal of Applied Physiology*.

[8] Shi X., Rehrer S., Prajapati P., Stoll S. T., Gamber R. G., Downey H. F. (2011). Effect of cranial osteopathic manipulative medicine on cerebral tissue oxygenation. *The Journal of the American Osteopathic Association*.

[9] Wyatt K., Edwards V., Franck L. (2011). Cranial osteopathy for children with cerebral palsy: A randomised controlled trial. *Archives of Disease in Childhood*.

[10] Raviv G., Shefi S., Nizani D., Achiron A. (2009). Effect of craniosacral therapy on lower urinary tract signs and symptoms in multiple sclerosis. *Complementary Therapies in Clinical Practice*.

[11] Hanten W. P., Olson S. L., Hodson J. L., Imler V. L., Knab V. M., Magee J. L. (1999). The effectiveness of CV-4 and resting position techniques on subjects with tension-type headaches. *Journal of Manual & Manipulative Therapy*.

[12] Cutler M. J., Holland B. S., Stupski B. A., Gamber R. G., Smith M. L. (2005). Cranial manipulation can alter sleep latency and sympathetic nerve activity in humans: a pilot study. *The Journal of Alternative and Complementary Medicine*.

[13] Martins W. R., Diniz L. R., Blasczyk J. C. (2015). Immediate changes in electroencephalography activity in individuals with nonspecific chronic low back pain after cranial osteopathic manipulative treatment: Study protocol of a randomized, controlled crossover trial. *BMC Complementary and Alternative Medicine*.

[14] Milnes K., Moran R. W. (2007). Physiological effects of a CV4 cranial osteopathic technique on autonomic nervous system function: A preliminary investigation. *International Journal of Osteopathic Medicine*.

[15] Nelson K. E., Sergueef N., Glonek T. (2006). The effect of an alternative medical procedure upon low-frequency oscillations in cutaneous blood flow velocity. *Journal of Manipulative and Physiological Therapeutics*.

[16] Downs S. H., Black N. (1998). The feasibility of creating a checklist for the assessment of the methodological quality both of randomised and non-randomised studies of health care interventions. *Journal of Epidemiology and Community Health*.

[17] Burt A., Morgan L., Petrinic T., Young D., Watkinson P. (2017). Usability evaluation methods employed to assess information visualisations of electronically stored patient data for clinical use: a protocol for a systematic review. *Systematic Reviews*.

[18] Cerritelli F., Lacorte E., Ruffini N., Vanacore N. (2017). Osteopathy for primary headache patients: Asystematic review. *Journal of Pain Research*.

[19] Cerritelli F., Ruffini N., Lacorte E., Vanacore N. (2016). Osteopathic manipulative treatment in neurological diseases: Systematic review of the literature. *Journal of the Neurological Sciences*.

[20] Licciardone J. C. (2017). Systematic review of comparative effectiveness and health economics research relating to osteopathic manipulative treatment. *Musculoskeletal Science and Practice*.

[21] Ernst E. (2012). Craniosacral therapy: A systematic review of the clinical evidence. *Focus on Alternative and Complementary Therapies*.

[22] Mulcahy J., Vaughan B. (2014). Sensations Experienced and Patients’ Perceptions of Osteopathy in the Cranial Field Treatment. *Evidence-Based Complementary and Alternative Medicine*.

[23] Alcantara J., Anderson R. (2008). Chiropractic care of a pediatric patient with symptoms associated with gastroesophageal reflux disease, fuss-cry-irritability with sleep disorder syndrome and irritable infant syndrome of musculoskeletal origin. *The Journal of the Canadian Chiropractic Association*.

[24] Evans S., Tsao J. C., Zeltzer L. K. (2008). Paediatric Pain Management: Using Complementary and Alternative Medicine. *Reviews in Pain*.

[25] Hundscheid H. W. C., Pepels M. J. A. E., Engels L. G. J. B., Loffeld R. J. L. F. (2007). Treatment of irritable bowel syndrome with osteopathy: Results of a randomized controlled pilot study. *Journal of Gastroenterology and Hepatology*.

[26] Attali T.-V., Bouchoucha M., Benamouzig R. (2013). Treatment of refractory irritable bowel syndrome with visceral osteopathy: Short-term and long-term results of a randomized trial. *Journal of Digestive Diseases*.

[27] Smith M., Clark K., Shi X., King H., Janig W., Patterson M. (2010). Manual medicine and the autonomic nervous system: assessing autonomic function in humans. *The Science and Clinical Application of Manual Therapy*.

[28] Henley C. E., Ivins D., Mills M., Wen F. K., Benjamin B. A. (2008). Osteopathic manipulative treatment and its relationship to autonomic nervous system activity as demonstrated by heart rate variability: a repeated measures study. *Osteopathic Medicine and Primary Care*.

